# Dual Immunomodulatory and Anti-Virulence Mechanisms of Curcumin Against *Salmonella enterica* Infection in Broilers: An Integrated Network Pharmacology and Molecular Docking Study

**DOI:** 10.3390/vetsci13040406

**Published:** 2026-04-20

**Authors:** Muhammad Jabbar, Mohamed Tharwat, Muhammad Younus, Muhammad Tariq, Abdallah A. Mousa, Saleh Alkhedhairi

**Affiliations:** 1 Cholistan Institute of Biological Sciences, Cholistan University of Veterinary and Animal Sciences, Bahawalpur 63100, Pakistan; 2 Department of Clinical Sciences, College of Veterinary Medicine, Qassim University, P.O. Box 6622, Buraidah 51452, Saudi Arabia; 3 Institute of Chemical Technology, Ural Federal University, Ekaterinburg 620002, Russia; myounuskhan636@gmail.com; 4 College of Animal Science and Technology, Nanjing Agricultural University, Nanjing 210095, China; tariq@stu.njau.edu.cn; 5 Animal Production Department, Faculty of Agriculture, Benha University, Benha 13736, Egypt; abdallah.alaa@fagr.bu.edu.eg; 6 Department of Medical Biosciences, College of Veterinary Medicine, Qassim University, P.O. Box 6622, Buraidah 51452, Saudi Arabia; s.alkhedhairi@qu.edu.sa

**Keywords:** broiler, curcumin, host–pathogen interaction, immunomodulation, molecular docking, network pharmacology, *Salmonella enterica*, virulence regulation

## Abstract

*Salmonella enterica* infection remains a major threat to poultry health and food safety, largely due to its ability to invade the intestinal epithelium, modulate host immunity, and persist intracellularly. *Curcumin*, a bioactive phytochemical, has shown promising antimicrobial and immunomodulatory potential; however, its precise molecular interplay with host and pathogen systems remains unclear. An integrated computational pipeline was applied, combining target prediction, host immune network construction, Salmonella virulence interaction analysis, STRING-based PPI mapping, KEGG/GO enrichment, and molecular docking validation. Host immune hub genes and Salmonella virulence regulators were identified, followed by docking of curcumin to key host (AKT1, STAT3, TNF) and pathogen proteins (invA, phoP, ssrB). Host network analysis revealed enrichment in the PI3K–AKT, NF-κB, FoxO, and IL-10 signaling pathways, indicating roles in epithelial protection, immune regulation, inflammation suppression, and antioxidant defense. Salmonella virulence hubs were primarily associated with epithelial invasion, Type III secretion, intracellular survival, and global virulence reg-ulation. Docking analysis demonstrated a strong binding affinity of curcumin toward AKT1 (−7.4 kcal/mol), STAT3 (−6.5 kcal/mol) and TNF (−5.8 kcal/mol), supporting host immunomodulation and epithelial protection. Simultaneously, curcumin showed notable affinity for phoP (−6.8 kcal/mol), invA (−6.3 kcal/mol), and ssrB (−5.8 kcal/mol), suggesting the potential suppression of virulence signaling, invasion machinery, and intracellular persistence. This integrated host–pathogen systems analysis demonstrates that curcumin exerts a dual regulatory effect by enhancing host immune protection while concurrently disrupting Salmonella virulence mechanisms. These findings provide mechanistic insight supporting curcumin as a promising natural therapeutic candidate for controlling Salmonella infection in broilers.

## Simple Summary

*Salmonella enterica* infection is a significant problem in poultry production and a significant cause of foodborne diseases in humans. Traditional methods of control are usually based on antibiotics, but rising resistance to antibiotics and expanding limitations on their use of antibiotics have led to an urgent necessity to find safe alternative methods. *Curcumin* is a natural product that is produced as a derivative of turmeric (*Curcuma longa*). In multiple animal studies, curcumin exhibited antimicrobial, antioxidant, and immune-promoting effects. This study employed complex computational methods, such as network pharmacology and molecular docking, to examine how curcumin can be used to manage *Salmonella* infection in broiler chickens. This study demonstrated that curcumin could interact with the key host immune proteins in regulating inflammation, antioxidants and the intestinal barrier. Concurrently, curcumin can also disrupt major Salmonella virulence proteins that cause bacterial invasion and survival within host cells. These results indicate that curcumin has the potential to exert dual action on poultry by boosting the strength of host defenses and reducing bacterial infection mechanisms. The findings of this study can be mechanistic in showing that curcumin is a promising natural alternative for enhancing the health of poultry and mitigating Salmonella infection in broiler production systems.

## 1. Introduction

*Salmonella enterica* is recognized as one of the most significant bacterial pathogens to animal production and human health across the globe [1,2]. Salmonella infection is one of the biggest challenges in poultry production systems because of its capability of colonizing the gastrointestinal tract in broiler chickens, staying contained in intestinal tissues, and contaminating poultry products that find their way into the human food chain [3]. A major reservoir of several Salmonella serovars, such as *Salmonella enteritidis* and *Salmonella typhimurium*, which are often linked to foodborne outbreaks in humans, is the broiler chickens [4]. After consumption, Salmonella may attach to the intestinal epithelial cells, infect host tissues via virulence factors, and alter host immune signal transduction pathways to persist in infection [5]. Such processes can damage intestinal barrier activity, cause inflammatory processes and destabilize the gut microbial state, in the end, affecting animal health and productivity [6]. In terms of public health, contaminated poultry meat and eggs are still one of the primary causes of human salmonellosis in the world, causing millions of infections each year and posing a major burden to the overall food safety systems across the world [7]. Therefore, poultry control strategies to prevent Salmonella colonization and infection have emerged as a primary concern for enhancing animal welfare, preventing zoonotic infections, and safeguarding poultry-based food items [8,9].

Control strategies for Salmonella infections vary considerably across geographical regions and production systems. In many developed regions, particularly within the European Union, preventive approaches such as vaccination, strict biosecurity measures, and surveillance programs are widely implemented, and the use of antibiotic growth promoters has been prohibited [10]. However, in several parts of the world, especially in intensive and developing poultry production systems, antibiotics have historically been used for disease control and growth promotion [11,12]. The widespread and, in some cases, uncontrolled use of antimicrobial agents has contributed to the rapid development of antimicrobial resistance (AMR), which is now recognized as a major global health concern [13]. Consequently, increasing regulatory restrictions and consumer demand for antibiotic-free poultry products have driven the search for safe and sustainable alternative strategies [14].

Curcumin, the main polyphenolic extract of Curcuma longa (turmeric), is one of the phytogenic compounds that have attracted a lot of scientific interest due to its broad spectrum of pharmacological actions [15,16]. Many studies have found, in both experimental and clinical studies, that curcumins have anti-inflammatory, antioxidant, antimicrobial and immunomodulatory properties [17]. Curcumin dietary supplements have been reported to increase growth rates, antioxidant means, as well as the immune response during various physiological stresses in the production of poultry [18]. Moreover, curcumin was reported to enhance the intestinal barrier integrity and inhibit the intestinal inflammation through signaling pathways that are linked to oxidative stress and inflammatory reactions [19].

Experimental evidence also shows that the supplementation with curcumin can inhibit Salmonella colonization and enhance the intestinal state of broiler chickens infected with Salmonella [20]. As an example, Leyva-Diaz et al. 2021 have shown that curcumin supplementation decreased the colonization of *Salmonella enteritidis* in broiler chickens and enhanced the intestinal permeability [20]. In another study, Hoffmann et al. 2026 showed that dietary curcumin lowers the infection of *Salmonella typhimurium* and enhances intestinal barrier health and microbiota balance in broilers [21]. Though previous studies suggest that curcumin can be very helpful in the control of bacterial infections in poultry, how exactly such protective effects are achieved at the molecular level is not fully understood yet [22,23].

One of the major limitations of past studies is that they tend to study either host immunity or bacterial inhibition alone, without considering the complicated interplay between host immunity defense systems and the virulence strategies of the pathogen [24]. The nature of infectious diseases involves dynamic host–pathogen interactions where both host regulatory mechanisms and bacterial virulence determinants are critical to the ultimate outcome of an infection [25,26]. Thus, the therapeutic potential of natural compounds like curcumin cannot be fully understood without an integrated system-level study, which has the capacity to analyze both the signaling networks of the host immune system and the virulence pathways of the pathogen in parallel [27].

Network pharmacology has become an effective systems biology platform for the study of the multi-target pharmacological activities of bioactive compounds. Network pharmacology combines bioinformatics databases, protein–protein interaction networks, pathway enrichment analysis and computational modeling to study the complex dynamics between compounds, genes, proteins and biological pathways, unlike the traditional pharmacological approaches that use single molecular targets [28,29]. The method is quite appropriate for phytochemicals like curcumin, which generally exert biological activities via various signaling pathways, like NF-KB, PI3K/AKT, STAT3 and NRF2 pathways, which regulate inflammation, immune responses, and oxidative stress [30]. Applying network pharmacology alongside molecular docking can offer a useful framework for the study of how a compound can be used to promote host immune defense, as well as disrupt pathogen virulence mechanisms, in the context of bacterial infections [31]. These dual-target therapeutic approaches are becoming viewed as potential alternatives to traditional antimicrobial approaches due to their potential to reduce selective pressure on antimicrobial resistance and enhance host resistance to infection [32].

The current work was intended to explore the dual mechanistic action of curcumin in relation to Salmonella enterica infection through an integrated host–pathogen computational system. In particular, the proposed study was designed to discover curcumin-related host immune and gut defense targets, describe pertinent virulence proteins in Salmonella pathogenicity, build protein–protein interaction networks to determine major regulatory centers, conduct Gene Ontology (GO) and Kyoto Encyclopedia of Genes and Genomes (KEGG) pathway enrichment, and molecular docking analysis to verify possible molecular interactions. The combination of host immune signaling networks with bacterial virulence determinants that form the basis of this study gives a comprehensive mechanistic understanding of how curcumin can be used to potentiate host immune defenses and antagonize Salmonella virulence mechanisms simultaneously, making it a useful natural therapeutic candidate to improve the health of poultry and the control of foodborne pathogens. The present study specifically focuses on broiler chickens due to their critical role as a primary reservoir of zoonotic Salmonella serovars and their direct contribution to foodborne transmission in humans. Broiler production represents one of the most intensive and economically significant sectors in poultry farming, where effective non-antibiotic intervention strategies are urgently required. Therefore, understanding host–pathogen interactions and identifying alternative control measures in broilers has direct implications for both animal health and public food safety.

## 2. Materials and Methods

### 2.1. Identification of Curcumin-Associated Target Proteins

Curcumin-associated target proteins were identified using well-established pharmacological databases. First, all known and predicted protein targets of curcumin were obtained from the Comparative Toxicogenomics Database (CTD; http://ctdbase.org; Mount Desert Island Biological Laboratory, ME, USA; accessed on 21 December 2025) and Swiss Target Prediction (https://www.swisstargetprediction.ch; Swiss Institute of Bioinformatics, Lausanne, Switzerland; accessed on 27 December 2026) by searching with the keyword “Curcumin [33].” The retrieved datasets were downloaded, and all protein entries were combined into a single list. Duplicate targets were removed to avoid redundancy, and each gene was cross-checked and standardized using UniProt (https://www.uniprot.org; UniProt Consortium, Cambridge, UK; accessed on 1 January 2026) to ensure correct gene nomenclature. The final list represented a comprehensive set of proteins potentially modulated by curcumin.

### 2.2. Collection of Broiler Host Immune and Gut Defense Genes

Broiler host immunity-related and gut defense-associated genes were collected through bioinformatics databases and published literature. The Gene Cards database (https://www.genecards.org; Weizmann Institute of Science, Rehovot, Israel; accessed on 4 January 2026) was searched using terms such as “broiler immunity,” “intestinal immunity,” “inflammation,” “oxidative stress,” and “gut barrier protection [34].” Relevant experimental and review studies were also consulted to ensure biological relevance. Genes associated with immune regulation, inflammatory response, antioxidant function, epithelial protection, and intestinal defense were included. These host defense genes were then compared with curcumin target genes, and the overlapping genes were considered potential host therapeutic targets of curcumin.

### 2.3. Identification of Salmonella enterica Virulence Proteins

Key virulence proteins of *Salmonella enterica serovar Typhimurium* were identified through literature-supported virulence research. Special emphasis was placed on proteins involved in epithelial invasion, the Type III secretion system (T3SS), intracellular survival mechanisms, global virulence gene regulation, and host interaction. Only functionally confirmed and biologically significant virulence regulators and effector proteins were included to ensure relevance to infection biology and pathogenicity. Although *Salmonella enterica serovar Typhimurium* was used as the primary model organism due to its well-characterized virulence mechanisms and availability of molecular data, key virulence determinants are highly conserved across major *Salmonella serovars*, including *Salmonella enteritidis*. Core virulence regulators such as invA, sipA, phoP, and ssrB are shared components of Salmonella pathogenicity islands (SPI-1 and SPI-2) and play similar roles in epithelial invasion, intracellular survival, and immune evasion across serovars. Therefore, the findings of this study can be reasonably extrapolated to *S. Enteritidis*, supporting broader applicability in poultry-associated Salmonella infections [35,36].

### 2.4. Construction of Protein–Protein Interaction (PPI) Networks

Protein–protein interaction (PPI) networks were constructed using the STRING database (https://string-db.org; SIB Swiss Institute of Bioinformatics, Lausanne, Switzerland; accessed on 6 January 2026) [37]. The overlapping host targets were uploaded into STRING, and the species was set to Homo sapiens with a confidence interaction score threshold of 0.4 to generate the host immune regulatory network. Similarly, selected Salmonella virulence proteins were uploaded into STRING with the species set to Salmonella enterica serovar Typhimurium and a confidence score of 0.5. Disconnected proteins were removed, and first shell interactors were included to obtain biologically meaningful interaction networks. All networks were downloaded for further topological and biological analysis.

### 2.5. Identification of Hub Proteins

The constructed STRING networks were imported into Cytoscape software (version 3.10.4; Cytoscape Consortium, San Diego, CA, USA) for network analysis [38]. Topological evaluation was performed to identify highly connected key nodes representing biologically important hub proteins. Ranking of nodes was based on network connectivity characteristics, and the top-ranking hub proteins were selected for subsequent functional pathway interpretation and validation analysis. These hub proteins were considered as primary molecular regulators.

### 2.6. Functional Enrichment Analysis

Functional enrichment analysis was performed to understand the biological roles of identified host and pathogen targets. For host proteins, Gene Ontology (GO) Biological Processes [39], and KEGG pathway enrichment [40] were obtained using STRING enrichment (https://string-db.org SIB Swiss Institute of Bioinformatics, Lausanne, Switzerland) analysis tools. Significantly enriched immune-related, inflammatory, survival, and stress response pathways were recorded. For Salmonella virulence targets, KEGG pathway enrichment was obtained from STRING, while GO Biological Process information was extracted using UniProt (https://www.uniprot.org; UniProt Consortium, Cambridge, UK) and QuickGO (https://www.ebi.ac.uk/QuickGO/annotations; EMBL-EBI, Hinxton, UK; accessed on 8 January 2026) due to limited GO annotation in STRING. Pathways were grouped according to immune signaling, epithelial barrier protection, oxidative stress response, bacterial invasion, virulence regulation, and intracellular survival.

### 2.7. Molecular Docking Analysis

Molecular docking was performed to evaluate the interaction of curcumin with key host and Salmonella proteins. The 3D structure of curcumin was obtained from the PubChem database (https://pubchem.ncbi.nlm.nih.gov; National Center for Biotechnology Information (NCBI), Bethesda, MD, USA; accessed on 20 January 2026), converted into a docking format, and energy minimized. Crystal structures of host proteins, including AKT1, STAT3, and TNF, were downloaded from the RCSB Protein Data Bank (PDB; https://www.rcsb.org; Research Collaboratory for Structural Bioinformatics (RCSB), Piscataway, NJ, USA; accessed on 20 January 2026) [41], whereas Salmonella proteins (invA, phoP, and ssrB) were retrieved from AlphaFold structural models (https://alphafold.ebi.ac.uk; EMBL-EBI, Hinxton, UK) when crystal structures were unavailable. All protein structures were prepared by removing water molecules, deleting heteroatoms, adding hydrogen atoms, assigning charges, and converting into PDBQT format.

Docking was performed using AutoDock Vina (version 1.2.x; The Scripps Research Institute, La Jolla, CA, USA) [42], where the grid box was centered around the active/binding site region of each protein. Docking was executed under optimized default settings, and the lowest binding energy conformation was considered the best binding pose. The resulting protein–ligand complexes were visualized using Discovery Studio Visualizer (version 25.1.0.24284; Dassault Systèmes, San Diego, CA, USA) [43]. Key interactions, including hydrogen bonding, hydrophobic interactions, electrostatic contacts, π–alkyl interactions, and stabilizing regions, were examined to provide biological meaning to the docking results. Binding affinities were recorded in kcal/mol for comparison.

## 3. Results

### 3.1. Identification of Common Curcumin—Host Immunity Targets

**Figure 1:**
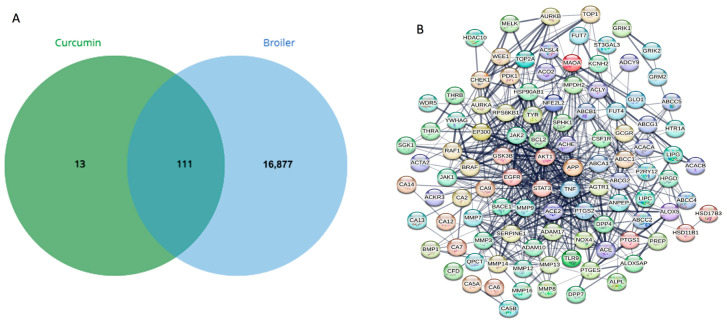
(**A**) Venn diagram representing overlapping target genes between curcumin-associated proteins and broiler immune/gut defense genes, showing 111 shared targets. (**B**) Overlapping genes from curcumin and broiler.

Integration of curcumin-associated target proteins with broiler immunity and gut defense genes resulted in 111 overlapping targets, indicating strong molecular crosstalk between curcumin signaling and host protective regulatory systems. These overlapping targets form the primary mechanistic basis through which curcumin may modulate immune protection, inflammatory balance, epithelial integrity, and stress resilience during Salmonella infection, and were carried forward for downstream network, enrichment, and docking analyses (Figure 1A,B).

### 3.2. Host Protein–Protein Interaction (PPI) Network Identifies Central Regulatory Hubs

**Figure 2:**
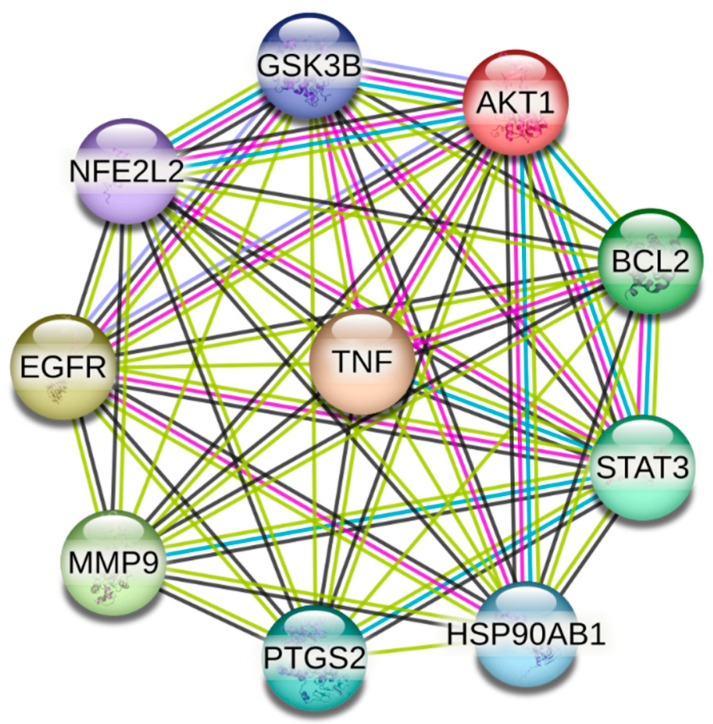
STRING-based PPI network of overlapping host targets (confidence 0.4), highlighting strong interconnectivity among immune regulatory proteins.

The STRING-derived host protein interaction network revealed a densely interconnected signaling landscape, highlighting significant biological coordination among identified immune targets. Cytoscape ranking identified AKT1, TNF, STAT3, PTGS2, GSK3B, EGFR, BCL2, HSP90AB1, MMP9, and NFE2L2 as dominant hub regulators displaying the highest connectivity. These hub proteins act as master regulators of immune signaling, cytokine balance, apoptosis suppression, antioxidant defense, and epithelial stability, indicating their key mechanistic importance in host defense regulation and potential modulation by curcumin (Figure 2) (Table 1).

### 3.3. GO Biological Processes Demonstrate Dominant Immune, Apoptotic, and Stress Regulation

**Figure 3:**
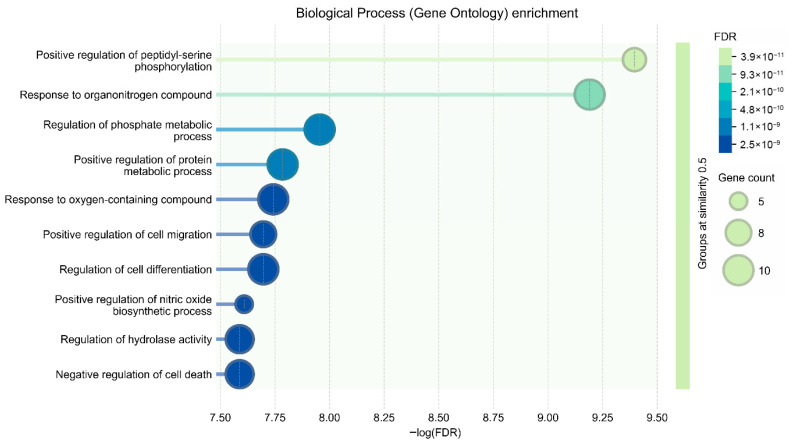
GO Biological Process enrichment bubble plot illustrating key immune, apoptotic, and stress-associated regulatory processes enriched among host targets.

GO Biological Process enrichment analysis demonstrated significant over-representation of biological processes related to immune modulation and cellular protection. The most prominent enriched processes included Interleukin-10 signaling, intrinsic apoptotic pathway regulation, BAD/BCL2-mediated mitochondrial survival signaling, growth factor receptor-associated pathways, and inflammasome-related responses. These enriched processes illustrate that curcumin-associated host targets predominantly regulate mechanisms responsible for inflammation control, epithelial survival, and immune homeostasis (Figure 3) (Table 2).

### 3.4. KEGG Pathway Analysis Confirms Immune-Protective and Survival-Supporting Signaling

**Figure 4:**
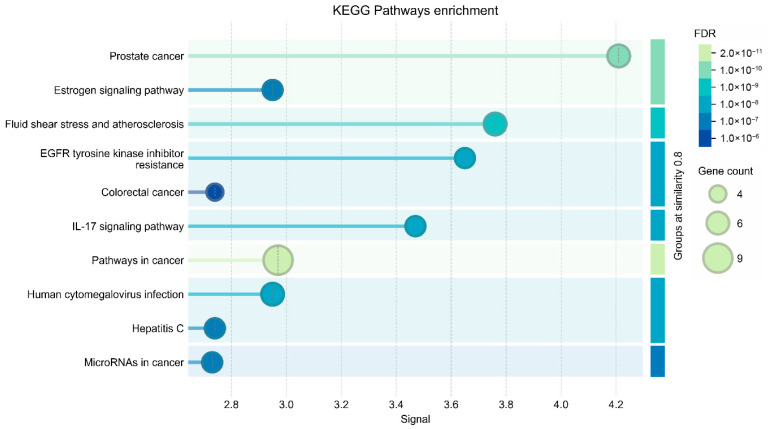
KEGG pathway enrichment bubble plot highlighting dominant immune regulatory and protective pathways.

KEGG enrichment analysis confirmed the dominance of protective host pathways, including NF-κB signaling, PI3K–AKT signaling, FoxO signaling, growth hormone signaling, and infection-related immune regulation pathways. These enriched pathways collectively emphasize the biological orientation of host hub targets toward limiting inflammatory injury, enhancing epithelial barrier survival, promoting antioxidant stability, and maintaining immune regulatory balance. These findings strengthen the mechanistic plausibility of curcumin as a host protective modulator (Figure 4) (Table 3).

### 3.5. Salmonella Virulence Network Reveals Central Invasion and Survival Regulators

**Figure 5:**
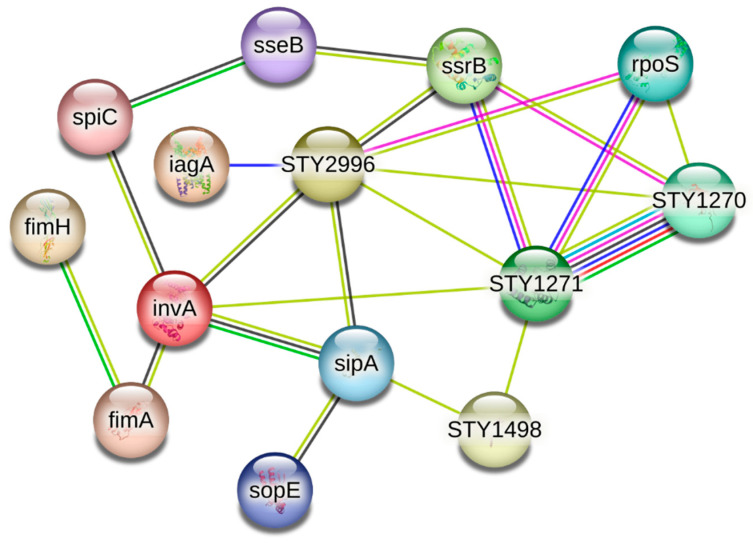
STRING interaction network of Salmonella enterica virulence proteins highlighting dominant infection-regulating hubs.

STRING-based interaction mapping of Salmonella enterica virulence proteins identified a strongly organized pathogenic network. The key virulence hubs identified included invA, hilD, phoP, sipA, and ssrB, functioning as major regulators of epithelial invasion, the Type III secretion activity, immune evasion, intracellular survival, and global virulence modulation. Their prominence confirms the accurate capture of critical determinants required for successful pathogenic establishment (Figure 5) (Table 4).

### 3.6. KEGG Pathways Confirm Core Invasion and Infection Establishment Strategies

**Figure 6:**
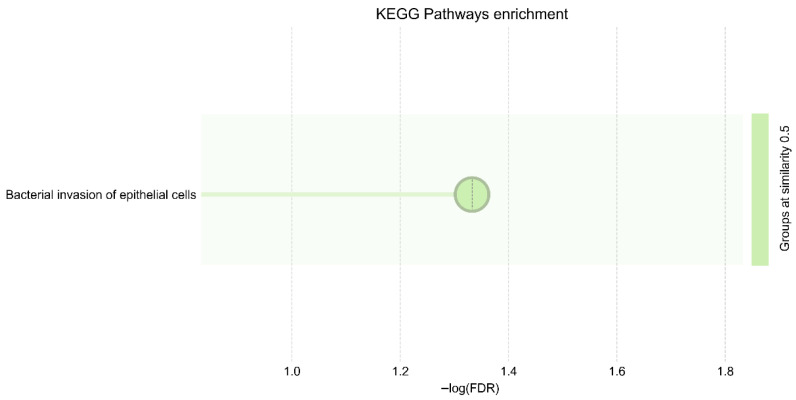
KEGG pathway enrichment map confirming enrichment in Salmonella invasion and infection pathways.

KEGG enrichment analysis revealed two highly significant pathways associated with Salmonella virulence: bacterial invasion of epithelial cells (FDR = 0.0145) and the Salmonella infection pathway (FDR = 0.0438). These pathways directly correspond to epithelial adherence, cytoskeleton manipulation, immune disruption, and infection establishment, validating the biological relevance of the selected virulence regulators (Figure 6) (Table 5).

### 3.7. Integrated Host–Pathogen Mechanistic Framework Suggests Dual Protective Role of Curcumin

**Figure 7:**
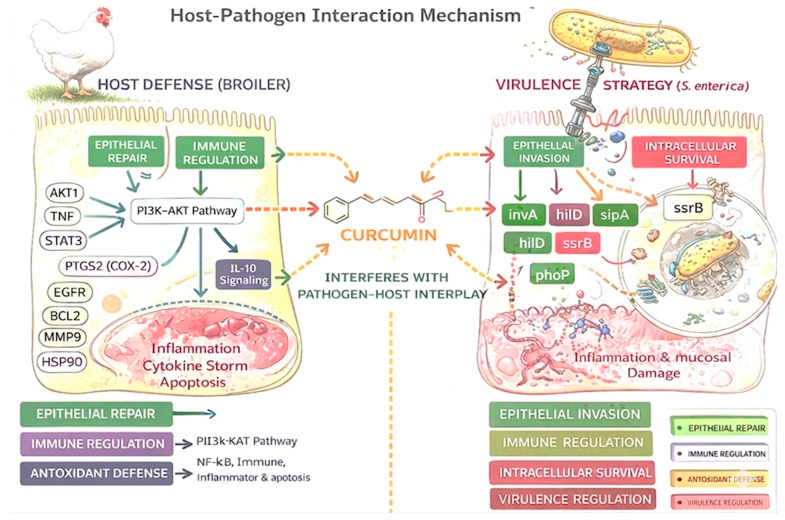
Integrated mechanistic model illustrating curcumin-mediated enhancement of host defense signaling and suppression of Salmonella virulence strategies. The epithelial invasion module includes invA, hilD, and sipA, representing coordinated regulation and execution of bacterial entry mechanisms.

Integration of host protective pathways and Salmonella virulence networks demonstrated an opposing biological interaction framework. While Salmonella promotes epithelial invasion, immune suppression, and intracellular persistence, host targets predominantly regulate immune balance, epithelial survival, and stress adaptation. Curcumin aligns structurally and functionally with host protective nodes while concurrently demonstrating potential interference with virulence regulators, establishing a plausible dual action therapeutic mechanism (Figure 7) (Table 6). In addition to previously highlighted virulence regulators, sipA was also incorporated into the integrated host–pathogen mechanistic model due to its critical role in actin cytoskeleton remodeling and the facilitation of epithelial invasion. Its inclusion further strengthens the representation of early-stage infection processes targeted by curcumin.

Table 6 summarizes the proposed mechanistic roles of curcumin derived from the integrated interpretation of network pharmacology analysis, pathway enrichment results, and molecular docking predictions, supported by relevant literature where available.

### 3.8. Molecular Docking Confirms Structural Basis of Dual Targeting Potential

**Figure 8:**
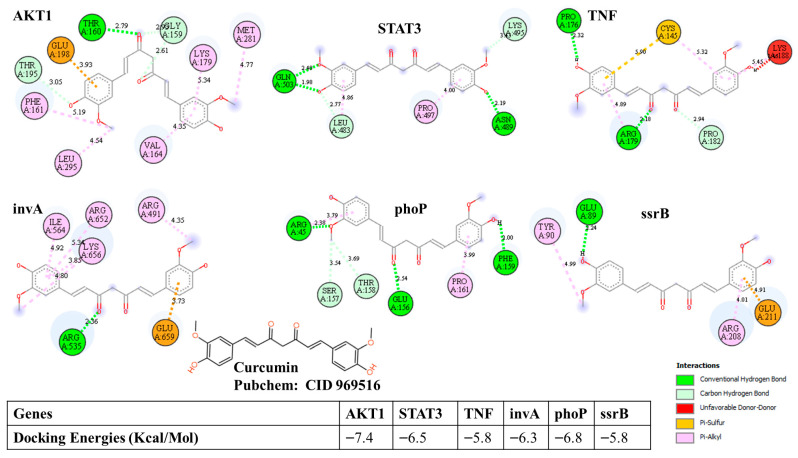
Representative docking interaction visualizations showing binding orientation and key interactions of curcumin with host and Salmonella proteins and molecular docking binding affinities.

Docking analysis demonstrated a strong binding potential of curcumin with key host and pathogen regulators. Among host proteins, the strongest affinities were observed for AKT1 (−7.4 kcal/mol), STAT3 (−6.5 kcal/mol), and TNF (−5.8 kcal/mol), indicating the structural potential for enhancing survival signaling, immune regulation, and inflammation modulation. Among Salmonella proteins, curcumin exhibited notable affinity toward phoP (−6.8 kcal/mol), invA (−6.3 kcal/mol), and ssrB (−5.8 kcal/mol), supporting interference with virulence regulation, invasion machinery, and intracellular persistence control (Figure 8).

## 4. Discussion

The present study suggests that curcumin may exert a dual protective mechanistic effect during Salmonella enterica infection by simultaneously strengthening host immune tolerance, epithelial protection, and oxidative defense, while concurrently suppressing key bacterial virulence determinants responsible for invasion, survival, and pathogenic persistence. Unlike previous curcumin studies that primarily emphasized performance improvement, biochemical stabilization, or antibacterial growth inhibition, this study provides integrated host–pathogen molecular mechanistic evidence, explaining how curcumin offers biological protection. Identification of 111 overlapping host targets with hub dominance of AKT1, STAT3, TNF, PTGS2, GSK3B, EGFR, BCL2, HSP90AB1, MMP9, and NFE2L2 indicates that curcumin primarily aligns with immune regulation, survival signaling, and stress adaptation networks [51]. These genes function as central regulatory nodes, meaning that even moderate modulation by curcumin can induce broad physiological stabilization.

AKT1, BCL2 and EGFR enrichment mechanistically explains the commonly reported improvements in intestinal integrity and epithelial survivability in curcumin-supplemented poultry. Activation of AKT1 promotes cell survival and reduces apoptosis [52], while BCL2 prevents epithelial cell death [53], and EGFR supports epithelial repair and mucosal barrier resilience [54]. Previous poultry studies reporting improved villus structure and intestinal restoration with curcumin supplementation [55] lacked a molecular explanation; our findings suggest that these improvements are driven by the coordinated activation of cell survival and epithelial growth pathways.

In our research a particularly important outcome was involvement of STAT3 and IL-10 signaling, indicating that curcumin does not simply “stimulate immunity,” but instead enhances immune tolerance and balanced immune homeostasis. STAT3-mediated IL-10 signaling helps prevent uncontrolled inflammation [56,57], a major contributor to tissue damage during infection. This aligns with [58], who reported that curcumin reduces the inflammatory burden and supports immune regulation.

Similarly, dominance of TNF, PTGS2 and NF-κB related pathways explains curcumin’s well-documented anti-inflammatory effects in poultry. Previous experiments demonstrated reductions in IL-6, TNF-α and systemic inflammation in curcumin-fed birds [50]. Another major mechanistic finding is the involvement of NFE2L2 (NRF2), the master regulator of cellular antioxidant defense [59,60]. In another study, it was reported that curcumin supplementation to broiler improved antioxidant capacity (higher SOD, catalase, and reduced oxidative stress markers) [51]. However, most publications attributed this only to curcumin’s chemical antioxidant nature. Our results showed that curcumin may biologically activate NRF2 signaling, thereby inducing endogenous antioxidant defense. This contributes to improved resilience against oxidative damage induced by Salmonella infection. Together, the host-related results explain that curcumin enhances controlled immunity, rather than excessive immunity, supports tissue protection, maintains mucosal stability, and promotes oxidative defense—changes that are biologically meaningful during infection stress.

The invA, phoP, hilD, ssrB, and sipA were observed as hub gene regulators, of Salmonella demonstrating that curcumin potentially interferes with pathogenic machinery rather than acting as a conventional antibacterial agent. invA and sipA are directly involved in host cell adhesion, invasion, and cytoskeletal manipulation [61], meaning their modulation can significantly reduce infection establishment. phoP and ssrB function as global virulence regulators [62], controlling stress adaptation, macrophage survival, secretion systems, and intracellular persistence. hilD coordinates the Type III secretion system activity, allowing Salmonella to manipulate host immune responses. Previous studies have reported a reduction in Salmonella counts when broilers were supplemented with curcumin [20]. Accordingly, sipA has been incorporated into the integrated mechanistic model to provide a more complete representation of epithelial invasion processes. Our findings support the newer biological paradigm that virulence suppression is more achievable and more sustainable than bacterial eradication. Unlike conventional antibiotics, targeting phoP/ssrB reduces disease potential without necessarily creating resistance pressure. While phytochemical-mediated virulence attenuation has been discussed in mammalian systems, poultry science lacked a clear molecular demonstration. Importantly, the virulence mechanisms evaluated in this study are conserved across major poultry-associated serovars, particularly *Salmonella enteritidis*, supporting the translational relevance of these findings in real-world poultry production systems. This work addresses that gap by linking curcumin to proteins controlling invasion and intracellular survival, strengthening the biological argument that curcumin limits pathogenic success rather than relying on bactericidal activity.

Docking results further support these conclusions by confirming physiochemically plausible interactions between curcumin and key Salmonella virulence proteins, complementing functional predictions and providing credible structural reinforcement. This combination of network pharmacology plus docking makes the virulence suppression concept scientifically stronger than hypotheses based only on gene expression or theoretical prediction. It should be noted that these interactions are derived from computational modeling and represent predictive insights rather than experimentally validated biological effects.

Although the present findings are based on computational predictions, they are supported by existing experimental evidence. Curcumin has been widely reported to modulate key host signaling pathways, including AKT, STAT3, and NF-κB, leading to reduced inflammatory responses and improved epithelial barrier integrity [63,64,65]. In addition, several studies have demonstrated that curcumin and related phytochemicals can interfere with bacterial virulence mechanisms, including the inhibition of Type III secretion systems and the suppression of virulence gene expression in Gram-negative pathogens [66,67,68,69,70]. These experimental observations provide biological support for the docking results obtained in this study and strengthen the plausibility of curcumin acting as a dual host-directed and anti-virulence agent.

Mechanistically, the predicted anti-virulence activity of curcumin may be suggested by its potential interference with key regulatory and effector components of the Salmonella pathogenicity system. Binding to regulators such as phoP and ssrB may disrupt global virulence gene expression and intracellular survival signaling, while interaction with invasion-associated proteins such as invA and sipA may impair the Type III secretion system function and cytoskeleton-mediated bacterial entry into host cells. Through these combined effects, curcumin is suggested to attenuate bacterial pathogenicity rather than exert direct bactericidal activity, representing a virulence-modulating strategy.

It is also important to consider the pharmacokinetic limitations of curcumin when interpreting these findings. Curcumin is a lipophilic compound with relatively low bioavailability and limited systemic absorption [71]. While its lipophilic nature allows interaction with cellular membranes and modulation of membrane-associated signaling pathways, its ability to reach intracellular targets may be significantly limited under physiological conditions [72]. However, previous studies have shown that curcumin can enter cells to a certain extent and that advanced delivery systems, such as nanoparticles, can enhance its intracellular bioavailability [73,74]. Therefore, the predicted interactions with intracellular targets should be interpreted with caution and may represent indirect or enhanced-delivery scenarios.

The principal novelty of this study is the demonstration of dual action: curcumin enhances host defense capacity while simultaneously compromising pathogen virulence performance. Most previous intervention strategies in poultry focused on either immune enhancement (vaccines, immunomodulators) or pathogen suppression (antibiotics, probiotics). curcumin may support both fronts, providing a biologically superior protective model.

It is important to emphasize that these findings are derived from computational modeling and predictive systems biology approaches and, therefore, represent hypothesis-generating insights rather than direct experimental evidence. Within this context, the results contribute to reshaping the conceptual understanding of curcumin from being merely an “antioxidant supplement” to a systems-level immune stabilizer and anti-virulence modulator. This study, therefore, bridges a critical gap in nutraceutical science, which has often been criticized for lacking mechanistic credibility.

Collectively, these findings explain why numerous poultry experiments report better health status, reduced inflammation, improved gut integrity, and a decreased Salmonella burden in curcumin-supplemented birds. This study provides a plausible molecular framework explaining how curcumin protects the host by activating key immune tolerance, epithelial survival, and oxidative resilience pathways, while concurrently weakening Salmonella invasion and survival strategies. This dual-mechanism framework supports curcumin as a scientifically meaningful, evolutionarily safer, and functionally relevant alternative strategy in poultry disease management.

Even though the current research study provides in-depth system-level understanding on the possible mechanisms of curcumin against *Salmonella enterica* infection, several limitations need to be noted. The results are founded on computational studies, such as network pharmacology and molecular docking predictions. Although these methods have useful mechanistic suggestions, experimental support using in vitro and in vivo experiments on broiler infection models is required to verify the suggested interactions and biological processes. The therapeutic potential of curcumin in the treatment of poultry diseases will be further elucidated in future research that incorporates transcriptomic, proteomic and experimental infection due to the integration of these methods. Additionally, this study does not explicitly address persistent Salmonella infections, such as those associated with reproductive tract colonization (e.g., the oviduct), which are critical for vertical transmission and long-term pathogen persistence in poultry systems.

## 5. Conclusions

This study provides a systems-level computational framework suggesting that curcumin may exert a dual-modulatory role during *Salmonella enterica* infection by potentially enhancing host immune defense while concurrently interfering with bacterial virulence mechanisms. The integration of network pharmacology, pathway enrichment, and molecular docking highlights plausible molecular interactions involving key host regulators and Salmonella virulence proteins. However, these findings are predictive in nature and require further validation through in vitro and in vivo experimental studies. Nonetheless, this work offers a mechanistic basis for future investigation of curcumin as a potential immunomodulatory and anti-virulence candidate in poultry health management. Future experimental validation using in vitro infection models and in vivo broiler trials will be essential to confirm the predicted interactions and further establish the translational potential of curcumin as an anti-virulence and immunomodulatory agent.

## Figures and Tables

**Table 1:** Top-ranked host hub proteins with node degree, interaction count, and inferred biological relevance.

Rank	Hub Gene	* Degree/Centrality	Interaction Count	Biological Relevance
1	AKT1	Highest	High	Cell survival, epithelial protection, immune regulation
2	TNF	Very High	High	Master pro-inflammatory cytokine
3	STAT3	Very High	High	Immune tolerance, IL-10 signaling
4	PTGS2	High	Moderate	Inflammatory mediator
5	GSK3B	High	Moderate	Immune–metabolic balance
6	EGFR	High	Moderate	Epithelial healing and repair
7	BCL2	Moderate	Medium	Anti-apoptotic survival
8	HSP90AB1	Moderate	Medium	Stress protein stabilization
9	MMP9	Moderate	Medium	Barrier damage inflammation
10	NFE2L2	Moderate	Medium	Master antioxidant regulator

* Degree/Centrality represents the relative connectivity of each node within the protein–protein interaction network, indicating its importance as a regulatory hub.

**Table 2:** Enriched GO Biological Processes with category, Pathway, Hub Genes and false discovery rate (FDR).

Category	Pathway	FDR	Hub Genes
Anti-inflammatory/Immune Defense	Interleukin-10 signaling	0.00031	STAT3, PTGS2, TNF
Apoptosis and Cell Protection	Intrinsic pathway for apoptosis	0.00043	STAT3, BCL2, AKT1
Cell Growth/Survival	Signaling by PTK6	0.00044	STAT3, EGFR, AKT1
Apoptosis Regulation	BH3-only proteins inactivate BCL2	0.0014	STAT3, BCL2
Cell Survival and Repair	PI3K/AKT signaling	0.0024	EGFR, GSK3B, AKT1
Stress Adaptation	AKT phosphorylates cytosolic targets	0.0033	GSK3B, AKT1
Mitochondrial Protection	BAD activation and mitochondrial translocation	0.0036	BCL2, AKT1
Inflammation Control	NLRP3 inflammasome	0.0038	APP, HSP90AB1
Signal Transduction Disorders	Diseases of growth-factor receptor signaling	0.0045	STAT3, EGFR, GSK3B, AKT1
Host Defense/Infection Response	Infectious disease	0.0046	EGFR, APP, GSK3B, HSP90AB1, AKT1

**Table 3:** KEGG pathways enriched in host targets with pathway name, involved genes, and FDR values.

KEGG Pathway	FDR	Key Hub Genes
NF-kappa B signaling	0.00014	PTGS2, BCL2, TNF
C-type lectin receptor signaling	0.00014	PTGS2, TNF, AKT1
T-cell receptor signaling	0.00014	GSK3B, TNF, AKT1
Yersinia infection pathway	0.00023	GSK3B, TNF, AKT1
Neurotrophin signaling	0.00018	GSK3B, BCL2, AKT1
FoxO signaling	0.00023	STAT3, EGFR, AKT1
Sphingolipid signaling	0.0002	BCL2, TNF, AKT1
Growth hormone signaling	0.0002	STAT3, GSK3B, AKT1
Relaxin signaling	0.00023	EGFR, MMP9, AKT1
Small cell lung cancer	0.00011	PTGS2, BCL2, AKT1

**Table 4:** Identified Salmonella virulence hub proteins and their primary functional roles in infection biology.

Hub Protein	GO Biological Process	Implication for Pathogenesis
invA	Invasion of host cells; type III secretion assembly	Key driver of intestinal invasion
hilD	Regulation of virulence gene expression	Master activator of SPI-1 pathogenicity island
ssrB	Regulation of intracellular survival	Promotes survival in host phagocytes
sipA	Host cytoskeleton modulation; invasion	Effector that facilitates entry into epithelial cells
phoP	Regulation of virulence and stress responses	Global control of virulence and adaptation

**Table 5:** KEGG enrichment summary for Salmonella virulence proteins with pathway name, involved genes, and FDR.

KEGG Pathway	FDR	Key Virulence Proteins	Biological Interpretation
Bacterial invasion of epithelial cells	0.0145	stpA, sipA	Indicates active engagement of invasion machinery and host cell entry mechanisms
Salmonella infection	0.0438	stpA, sipA	Reflects global mechanisms associated with infection establishment and virulence expression

**Table 6:** Core role of curcumin.

Mechanism	Molecular Support	References
Reduces inflammation	decreases NF-κB, TNF, PTGS2	[44]
Enhances antioxidant defense	activates NRF2	[45]
Supports epithelial barrier	EGFR, AKT1 pathways	[46,47]
Prevents apoptosis damage	BCL2 activation	[48]
Maintains immune balance	IL-10/STAT3 signaling	[49]
Inhibits epithelial invasion	invA, sipA	[50]

## Data Availability

The original contributions presented in this study are included in the article. Further inquiries can be directed to the corresponding author(s).

## Abbreviations

The following abbreviations are used in this manuscript:

AKT1	Protein Kinase B
AMR	Antimicrobial Resistance
BCL2	B-Cell Lymphoma
CTD	Comparative Toxicogenomics Database
EGFR	Epidermal Growth Factor Receptor
GO	Gene Ontology
HSP90AB1	Heat Shock Protein 90 Alpha Family Class B Member 1
IL-10	Interleukin-10
KEGG	Kyoto Encyclopedia of Genes and Genomes
MMP9	Matrix Metalloproteinase 9
NF-κB	Nuclear Factor Kappa-Light-Chain-Enhancer of Activated B Cells
NRF2 (NFE2L2)	Nuclear Factor Erythroid 2-Related Factor 2
PDB	Protein Data Bank
PI3K–AKT	Phosphoinositide 3-Kinase–Protein Kinase B Signaling Pathway
PPI	Protein–Protein Interaction
PTGS2	Prostaglandin-Endoperoxide Synthase 2
ROS	Reactive Oxygen Species
STAT3	Signal Transducer and Activator of Transcription 3
STRING	Search Tool for the Retrieval of Interacting Genes/Proteins
T3SS	Type III Secretion System
TNF	Tumor Necrosis Factor
